# Experimental study on the galloping characteristics of single ice-coated transmission lines under oblique flows

**DOI:** 10.1038/s41598-023-32393-y

**Published:** 2023-03-30

**Authors:** Mingyang Gao, Zhaoqing Chen, Jin Su, Ning Su, Chuncheng Liu, Jinlong Zhang, Huiru Chen

**Affiliations:** 1 School of Civil Engineering and Architecture, Northeast Electric Power University, Jilin, 132012 China; 2 Key Lab of Electric Power Infrastructure Safety Assessment and Disaster Prevention of Jilin Province, Northeast Electric Power University, Jilin, 132012 China; 3 Heilongjiang Electric Power Design Institute CO., LTD, China Energy Engineering Group, Harbin, 150078 China; 4 Tianjin Research Institute for Water Transport Engineering, Ministry of Transport, Tianjin, 300456 China; 5 Jiangmen Power Supply Bureau, Guangdong Power Grid Corporation, Jiangmen, 529000 China

**Keywords:** Civil engineering, Electrical and electronic engineering

## Abstract

Galloping of ice-coated transmission lines is occasionally observed under oblique wind directions. However, most current investigations on the galloping mechanisms are for flow perpendicular to the span of the transmission lines. In order to address this gap, this research studies the galloping characteristics of ice-coated transmission lines under oblique flows based on wind tunnel tests. The wind-induced displacement of an aero-elastic iced-coated transmission line model was measured with a noncontact displacement measurement equipment in a wind tunnel at different wind speeds and directions. The results show that galloping is characterized by elliptical trajectories and negative damping, which is more likely to occur under oblique flows than the direct flow (0°). At 15° wind direction, a galloping in vertical direction was observed at wind speeds above 5 m/s. At 30° wind direction, galloping was observed over the entire range of the tested wind speeds. Moreover, the galloping amplitudes under oblique flows are observed to be larger than that at the direct flows. Consequently, when the wind direction between the major winter monsoon azimuth and the lateral direction of transmission line route is between 15° and 30°, appropriate anti-galloping devices are highly recommended in practice.

## Introduction

The galloping of an ice-coated transmission line is characterized as a low-frequency, large-amplitude, self-excited vibration under wind excitation. Galloping may cause fatigue and damage of transmission lines and wiring fittings. Moreover, it may even cause the collapse of the transmission tower^1^. The galloping mechanism of ice-coated transmission lines is a hot-spot issue in disaster prevention and mitigation of transmission line systems. Analytical methods, numerical simulations, field measurements, and wind tunnel tests are the major methods to study the galloping mechanism of ice-coated transmission lines.

In terms of the analytical theories of galloping, Den Hartog^2^ proposed a simplified single-degree-of-freedom quasi-steady model to consider the vertical vibration. Nigel^3^ proposed a torsional excited galloping mechanism by extending Den Hartog’s theory. Yu et al.^4,5^ developed an inertia-coupling mechanism. It is indicated that, due to the change in the attack angle caused by the eccentric inertia resulted from icing, the lift force provides positive feedback to the lateral vibration, thus, forming a substantial galloping phenomenon. By using the abovementioned analytical methods, the occurring conditions and influencing factors of galloping are discussed. Consequently, the understanding of galloping was enhanced for practice. Liu et al.^6^ added an external excitation load to the governing equation of iced transmission lines based on the condition of stable wind, thus establishing a new forced self-excited vibration model for galloping. Liu et al.^7^ analyzed the accuracy of approximate solutions obtained by the perturbation method on the galloping equations. Based on these investigations, theoretical design criteria which are helpful for restraining or eliminating the galloping of transmission lines were proposed. However, in practical engineering, galloping even occurs occasionally beyond the condition calculated based on classical theories, indicating that more complex realistic situations should be further considered.

Massive attempts are performed to simulate the galloping process of transmission lines using numerical methods. Based on the spatial curved beam theory, Yan et al.^8,9^ established two kinds of iced conductor galloping models, namely, a finite curved beam model and a mixed model, to simulate the galloping of transmission lines. Consequently, a formula for the critical wind speed was proposed. Wu et al.^10^ used the commercial CFD software FLUENT to simulate the air flow around two bundle conductors. A numerical simulation method for wake oscillation was proposed, which is validated by the wind tunnel data. The results have shown that, when galloping occurs, the trajectory of a sub-conductor is close to the horizontal ellipse. Meynen et al.^11^ simulated the energy input characteristics of a single conductor numerically by solving a two-dimensional laminar simple harmonic oscillation cylinder problem. Clunia et al.^12^ analyzed the fatigue life of a transmission line by numerical simulations in laminar and turbulent flow. Desai et al.^13^ proposed a cable element with torsional degree-of-freedom to simulate an iced conductor. Xiong et al.^14^ conducted modal analysis on iced conductors and determined their galloping performance by using a three-dimensional curved beam model. Zhang et al.^15^ performed an aero-elastic test of four bundled conductors and analyzed the vibration modes considering different types of insulators. Based on these investigations, the galloping process of transmission lines can be numerically reproduced. The interactions between wind and transmission lines were discussed. However, due to the complexity and deficiencies in calculating accuracy and efficiency, their applications to practical engineering were limited.

Some investigations are based on field observations of the galloping of realistic transmission lines and put forward a series of measures to prevent galloping. Gurung et al.^16^ introduced a multi-channel modal analysis method to monitor the wind-induced vibration of the Tsuruga test line. In this approach, the random decrement method (RDM) and eigen system realization algorithm were adopted to identify the galloping. The galloping characteristics, such as the vibration mode, the interaction between translation and rotation, the vibration envelope, and the influence of the geometric shape, were discussed. Hung et al.^17^ performed eigenvalue analysis on the field-measured data, combined with the finite element method, to analyze the large-amplitude gust response characteristics of overhead transmission lines. The data of average wind speed, wind direction, turbulence intensity, and the root of mean square (RMS) values, the power spectral density (PSD) functions of wind-induced responses were analyzed to identify the types of wind-induced vibration. With eigenvalue analysis, gust response analysis was performed in the frequency domain to classify the vibration types of different transmission lines. Dyke and Laneville^18^ observed the in-situ wind-induced responses of a D-shaped iced single conductor and three bundled conductors on the high-voltage overhead transmission lines in Walaney. It is found that the azimuth of wind may strongly affect the galloping amplitude of a conductor. Based on these studies, a series of suggestions have been proposed to prevent or limit galloping. However, it is difficult to control the wind speed, turbulence, wind direction and other environmental factors. It is also challenging to explain the galloping mechanism of transmission lines through field measurements.

To investigate the influences of different factors (such as wind speed, angle of attack, shape of ice cover, etc.) on galloping, wind tunnel experiments are carried out on rigid and aero-elastic models. For example, Guo et al.^19,20^ performed an experimental study on the galloping characteristics of ice-coated conductor. Based on the aerodynamic coefficient obtained through a force measurement wind tunnel test, the accuracy between the theoretical formula and experimental results was revealed. A new method for protecting ice-coated conductor from galloping was provided. Chabart and Lilien^21^, Zdero et al.^22^, Li et al.^23^, Lou et al.^24,25^, and Zhou et al.^26^ conducted wind tunnel tests with single or multi-bundled (four-, six-, or eight-bundled) iced conductor models supported by springs. The galloping mechanism of transmission lines are enriched through these investigations which are valuable for the prevention and control of galloping. However, in most existing wind tunnel studies, only one or a few ice-coated conductor units are adopted as the research object. The influences of wind direction on galloping characteristics are rarely considered.

In addition, the relationship between the natural frequency ratio of the conductor and the galloping characteristics was investigated to further reveal the galloping mechanisms. Yan et al.^27^ proposed a finite element method for calculating the galloping response of ice-coated transmission. In steady-state and random wind fields, the galloping characteristics of transmission lines with and without internal resonance were discussed. A parameter is proposed to identify the galloping pattern and the coupled motion caused by the internal resonance. Liu et al.^28^ established a mechanical model for three-degree-of-freedom ice-coated transmission lines. The influence of conductor tension on the in-plane and out-of-plane vibration frequencies and the critical wind speed for galloping are addressed.

In the existed investigations, the in-plane and out-of-plane modes for single-span or multi-span transmission lines under different wind speeds and angles of attack are considered. In most of the field observation, when the iced transmission line is galloping, the direction of the incoming wind is rarely completely perpendicular to the direction of the transmission line span. Therefore, the influences of wind directions on the galloping deserve a special attention. However, the experimental investigation of oblique flows on galloping characteristics is still lacking. Moreover, most of the existing studies only consider the motion in the plane consisting of along-wind and vertical directions. The excitations of the in-plane vibration of the transmission line on the galloping are rarely discussed. Although some investigations on the mechanism of coupling between in-plane and out-of-plane vibrations of the transmission lines are carried out theoretically or numerically, experimental validations are still required.

In order to address these gaps, this paper aims to explore the galloping characteristics under oblique flows in detail based on wind tunnel tests. A noncontact, multipoint, synchronous displacement measurement equipment was used to measure the wind-induced responses of a series of aero-elastic ice-coated transmission line models in a wind tunnel. The wind-induced galloping characteristics (such as statistics of vibration, power spectral densities, vibration trajectories and damping ratios) are investigated. Particularly, the influences of oblique flows (15° and 30°) on the galloping response characteristics are addressed. The vibration characteristics of the wind vibration response of the ice-coated single conductor at different wind directions were compared. Finally, the influences of the oblique wind on the galloping response characteristics were clarified for practical reference.

## Wind tunnel test on an aero-elastic model

The experiment was conducted in the boundary layer wind tunnel laboratory in Tianjin Research Institute for Water Transport Engineering. The wind tunnel is a horizontal open circuit wind tunnel. The dimension of the test section is 4.4 m (width) × 2.5 m (height) × 15 m (length). The test wind speed can be controlled continually within a maximum of 30 m/s.

### Design and manufacture of the aero-elastic model

To ensure that the wind-induced response observed in the wind tunnel laboratory can be accurately converted into the prototype scale, similarity criteria should be considered in the design of the aero-elastic model. According to the similarity analysis in wind tunnel test of tower line system^29^, it is generally necessary to consider the similarity of the flow field, geometries, and structural vibration characteristics. On this basis, the scale ratios of the aero-elastic model can be determined. The ice-coated transmission line model with both ends fixed was designed. In general, three basic scale ratios, namely, the geometric* λ*_*l*_, wind speed *λ*_*U*_, and density ratios *λ*_*ρ*_, can be determined independently. In this study, the span of the aero-elastic model and prototype are 3.4 m and 68 m, respectively. Therefore, the geometric scale ratio *λ*_*l*_ is determined as 20:1. The wind speed *λ*_*U*_ and density ratio *λ*_*ρ*_ are set as 1:1, respectively. The other scale ratios, such as sag ratio *λ*_*h*_, quality ratio *λ*_*m*_, frequency ratio *λ*_*f*_, and elastic modulus ratio *λ*_*E*_, can be derived through the proportional relationship formulas listed in Table 1. However, not all the theoretical scale ratios can be satisfied at the same time. For example, the actual scale ratio of the mass ratio is quite different from the theoretical scale ratio. If the scaled transmission line and ice model can be made of aluminium alloy ice materials, respectively, the theoretical scale ratio would be consistent with the actual scale ratio. However, no aluminium alloy transmission line model with such a small cross section can be found in nature, and it is impossible to simulate the icing section model with real ice in the normal temperature in the wind tunnel. Therefore, wire rope and gypsum were used to make the scaled transmission line and ice models, which resulted in the density of the scale model to be larger than the actual one and the response tested in the experiment to be smaller than the actual one. However, the scaled model with deviation of mass ratio would not cause the disappearance of typical galloping characteristics such as elliptical trajectory and negative aerodynamic damping during the galloping process. Therefore, the scaled model with deviation of mass ratio is still used to study the influence of wind direction change on the galloping conditions in this study.

**Table 1:** Scaling ratios for various physical parameters of the aero-elastic model.

Physical quantity	Proportional relationship	Theoretical scale ratio	Actual scale ratio	Values for the test model
Span (*l*)	$\lambda_{l} = l_{{\text{p}}} /l_{{\text{m}}}$	20:1	20:1	3.4 m
Sag (*h*)	$\lambda_{h} = h_{{\text{p}}} /h_{{\text{m}}}$	10:1	10:1	0.34 m
Wind speed (*U*)	$\lambda_{U} = U_{{\text{p}}} /U_{{\text{m}}}$	1:1	1:1	4–10 m/s
Density (*ρ*)	$\lambda_{\rho } = \rho_{{\text{p}}} /\rho_{{\text{m}}}$	1:1	1:1	1.293 kg/m^3^
Quality (*m*)	$\lambda_{m} = \lambda_{\rho } \lambda_{l}^{2}$	4000:1	250:1	0.1145 kg
Frequency (*f*)	$\lambda_{f} = \lambda_{U} /\lambda_{l}$	1:20	1:20	–
Elastic modulus (*E*)	$\lambda_{E} = \lambda_{\rho } \lambda_{U}^{2}$	1:1	1:1	73.0 GPa

The scaling ratios for various physical parameters of the aero-elastic are listed in Table 1. In the table, the subscripts “p” and “m” indicate the prototype and model scales, respectively.

The transmission line in prototype scale is assumed to be with a span of 68 m, a maximum sag of 3.4 m. The model of transmission conductor is selected as LGJ-240/30, which is with a diameter of 30 mm. The diameter of the transmission line is 21.6 mm, and the mass per unit length is 922.2 kg/km. According to the similarity theory, a scaled model with above-listed similarity ratios is designed. The dimensions of the aero-elastic model are determined according to the scale ratios. The resulting dimensions of the prototype and the model are shown in Table 2.

**Table 2:** Dimensions of prototype and model scales.

Physical quantity	Symbol	Prototype	Model
Span (m)	*l*	68	3.4
Sag (mm)	*h*	3400	400
Diameter of transmission line (mm)	*d*	30	1.5
Icing diameter (mm)	*M*	50	24
Height of hanging point (m)	*P*	34	1.7
Wind speed (m/s)	*U*	4–10	4–10

**Figure 1:**
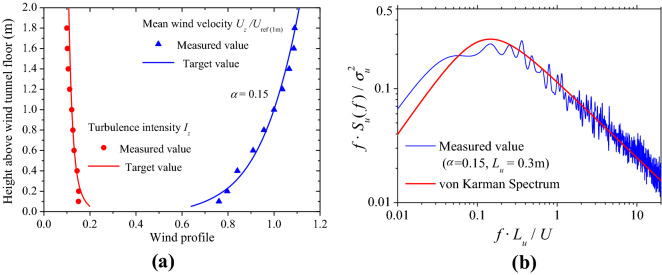
Atmospheric boundary layer simulation results. (**a**) Mean velocity and turbulence intensity profiles; (**b**) Wind velocity power spectrum.

Considering that the galloping of transmission lines is mostly observed in the open terrain areas, a standard B-type terrain in GB50009-2012 is used in the simulating the atmospheric boundary layer. In this experiment, wedge and rough elements are used to simulate the wind field of the wind profiles with an exponential index *α* = 0.15. The simulated wind field is measured before the test to verify whether it meets the target values. The comparison between the measured and targeted wind profiles are shown in Fig. 1, where *U*_*z*_ and *U*_*ref*_ are the mean wind speed at height *z* and reference height, respectively. *I*_*z*_ is the turbulence intensity at height *z*, *S*_*u*_(*f*) is the power spectrum of the fluctuating wind velocity, *f* is the frequency,* L*_*u*_ is the turbulence integral scale and *σ*_*u*_ is the standard deviation of the wind speed *U*. The results show that the mean wind speed profile, turbulence intensity profile and wind speed power spectrum simulated in the wind tunnel agree well with the simulation targets from the codes.

**Figure 2:**
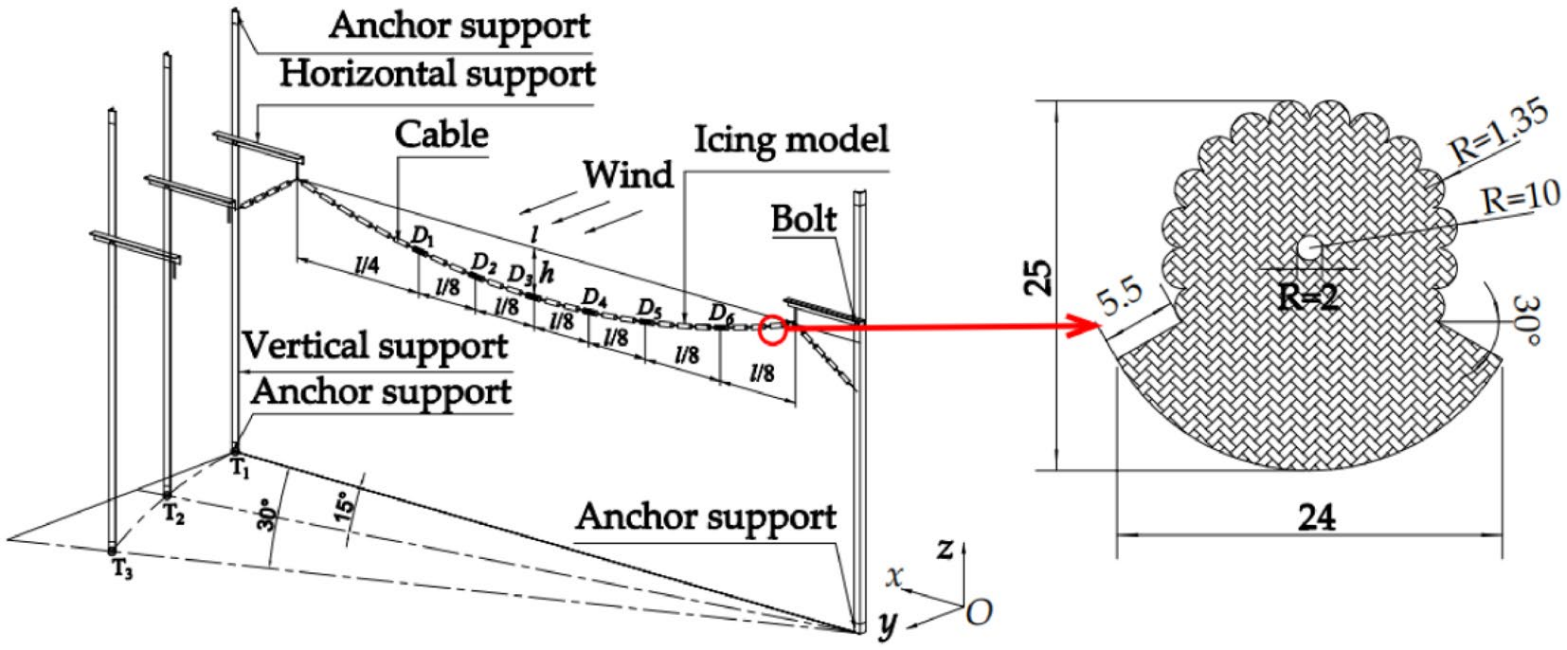
Schematic diagram of model installation, wind direction and fan-shaped icing section.

**Figure 3:**
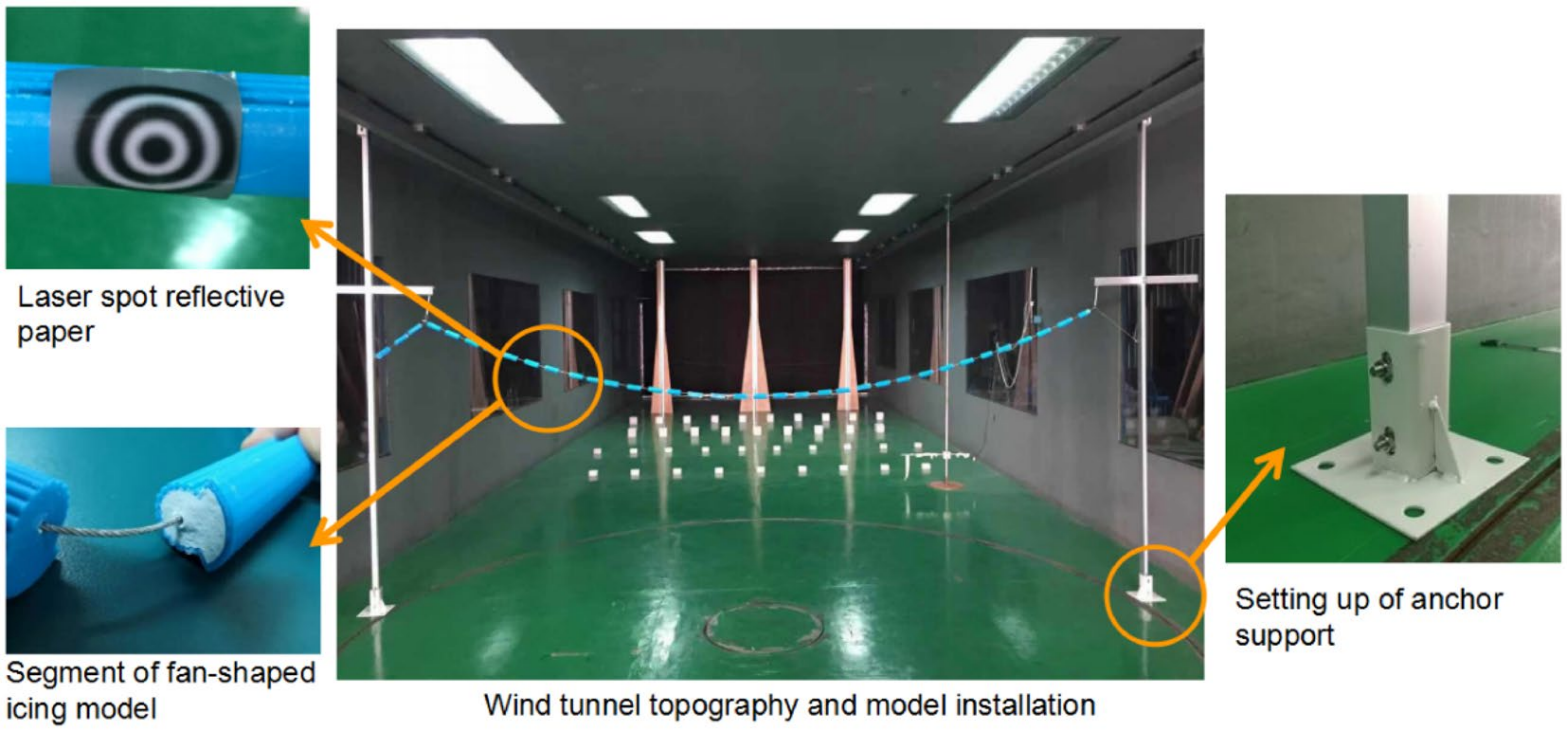
Photos of wind tunnel test with details of model configurations and installations.

The aero-elastic test model of the ice-coated transmission line was adopted as a fan-shaped ice segment model. The dimension of the fan-shaped ice segment model is shown in Fig. 2. Each segment was with a length of 70 mm. The fan-shaped ice segment models were manufactured with PLA (Poly Lactic Acid) material and connected by a 1.5 mm-diameter steel wire with a Young’s modulus of 165,000 MPa. The fan-shaped icing segments were affixed to the wire by gypsum filling at an equal interval of 15 mm on the wire, as shown in Fig. 3. The total weight of the icing model and the transmission line is 1.58 kg. Both ends of the wire were connected to a tension spring fixed at the columns as equivalent towers to simulate the support of transmission tower. Each column is installed by anchor brackets between the floor and ceiling of the wind tunnel. The photo of overall test model is shown in Fig. 3.

### Measurement scheme and test cases

The wind speed measurement is based on a Cobra probe arranged 2.5 m in the upstream direction of the model. The wind speed time histories of three directions (x, y, z) are collected at the height of the hanging point. The sampling frequency is 1024 Hz. A noncontact, multipoint displacement detection system was used to measure the wind-induced displacement time histories. The displacement measurement points are shown in Fig. 3. Laser reflective paper is installed at each measurement point to capture the displacement data. The wind direction angle can be adjusted by moving and rotating the equivalent tower, as shown in Fig. 2, where T1, T2, and T3 are tower positions corresponding to the wind directions of 0°, 15°, and 30°, respectively. The coordinate system is defined as follows, the x-direction represents the direction along the transmission line, the y-direction represents the out-of-plane direction, and the z-direction represents the in-plane direction.

In the test procedure, 0°, 15°and 30° wind directions are considered. For each wind direction case, the wind speeds ranged from 4 to 10 m/s at an interval 1 m/s are set as subcases. For each subcase, a total of 6 measurement points are arranged, named as D_*i*_ (*i* = 1, 2, …, 6), as shown in Fig. 2. The wind-induced displacement of the points, and the reference wind speed are measured during the wind tunnel test.

### Data processing and analysis

In this paper, the modal vibration frequencies, time histories, power spectra, vibration trajectories, and damping ratios are analyzed to determine the occurrence and type of galloping. The data processing and analysis methods are described as follows.

Usually, the mean and standard deviation values of the displacement time history are used to quantify the time-averaged deformation and vibration amplitude of the structure^17,29^. The evaluation methods are displayed as the following equations:

1 $$\overline{d}_{i} = \frac{1}{N}\sum\limits_{j = 1}^{N} {x_{i} (t_{j} )}$$

2 $$\sigma_{i} = \sqrt {\frac{1}{N - 1}\sum\limits_{j = 1}^{N} {[x_{i} (t_{j} ) - \overline{d}_{i} ]^{2} } }$$

where* x*_*i*_(*t*_*j*_) is the wind-induced displacement time-history of point D_*i*_ (*i* = 1, 2, …, 6) at time *t*_*j*_ (*j* = 1, 2, …, *N*), with *N*, which equals to 50,000, representing the total number of the sampling data.$\overline{d}_{i}$ is the mean value of wind-induced displacement of point D_*i*_ (*i* = 1, 2, …, 6). *σ*_*i*_ is the standard deviation value of wind-induced displacement of point D_*i*_ (*i* = 1, 2, …, 6), representing the magnitude of deviating from the equilibrium position during the vibration, which is also denoted as the amplitude in the followings.

The solution method to the aerodynamic damping of the transmission line in this paper is described as follows. Firstly, the wind-induced displacement time histories are filtered with the Butterworth filter for the targeted frequency band around the dominant vibration frequency. Subsequently, using Random Decrement Technique (RDT)^30,31^, the free vibration curves are obtained. Finally, the damping ratios in different modes are identified by the exponential enveloped of the free vibration curves obtained through Hilbert transform.

**Figure 4:**
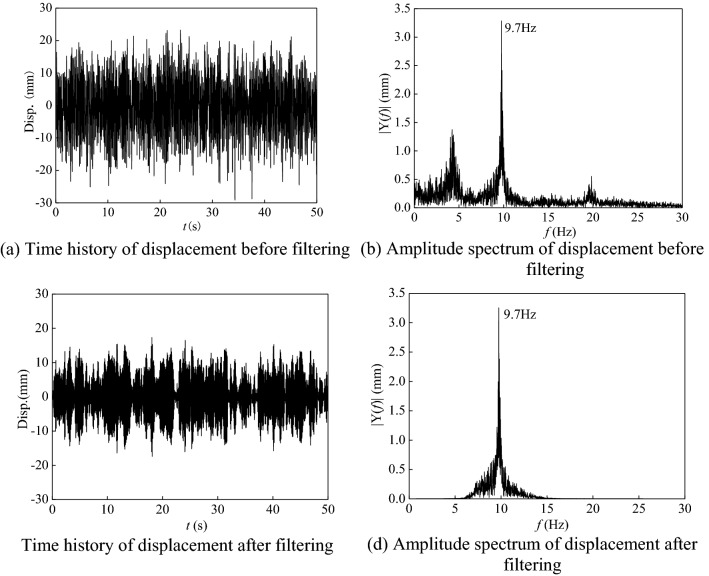
Time histories of displacement and amplitude spectrum before and after filtering.

The displacement time-histories processed by conventional filtering techniques often show abrupt changes in response spectrum amplitude and energy decay, which would affect the accuracy of damping ratio identification. Therefore, this paper adopts Butterworth filter in extracting the modal vibration results. Figure 4 shows the displacement response and power spectrum of the response before and after filtering. In the illustration, the filtering band is 5–15 Hz. It can be observed that the energy components in the unconcerned frequency band can be well filtered out by using this method. There is no frequency shift in the power spectrum of the displacement response after filtering. The amplitude of the power spectrum for the concerned frequency band is not decayed.

Random Decrement Technique (RDT) is used to extract the free decay vibration signal of the structure from the random vibration response signal of the ice-covered transmission line model. Hilbert transform is used to estimate the damping ratio of the free vibration curve identified by the RDT.

It is generally believed that the total displacement response *X*_*T*_ of the structure under zero-mean stationary random process excitation is superimposed by three different response components as shown in Eq. (3).

3 $$X_{T} = X_{{X_{0} }} + X_{{V_{0} }} + X_{F}$$

where *X*_*X*0_ represents the response caused by the initial displacement X_0_; *X*_*V*0_ denotes the response caused by the initial velocity *V*_0_ and *X*_*F*_ denotes the response caused by an external force *F*.

**Figure 5:**
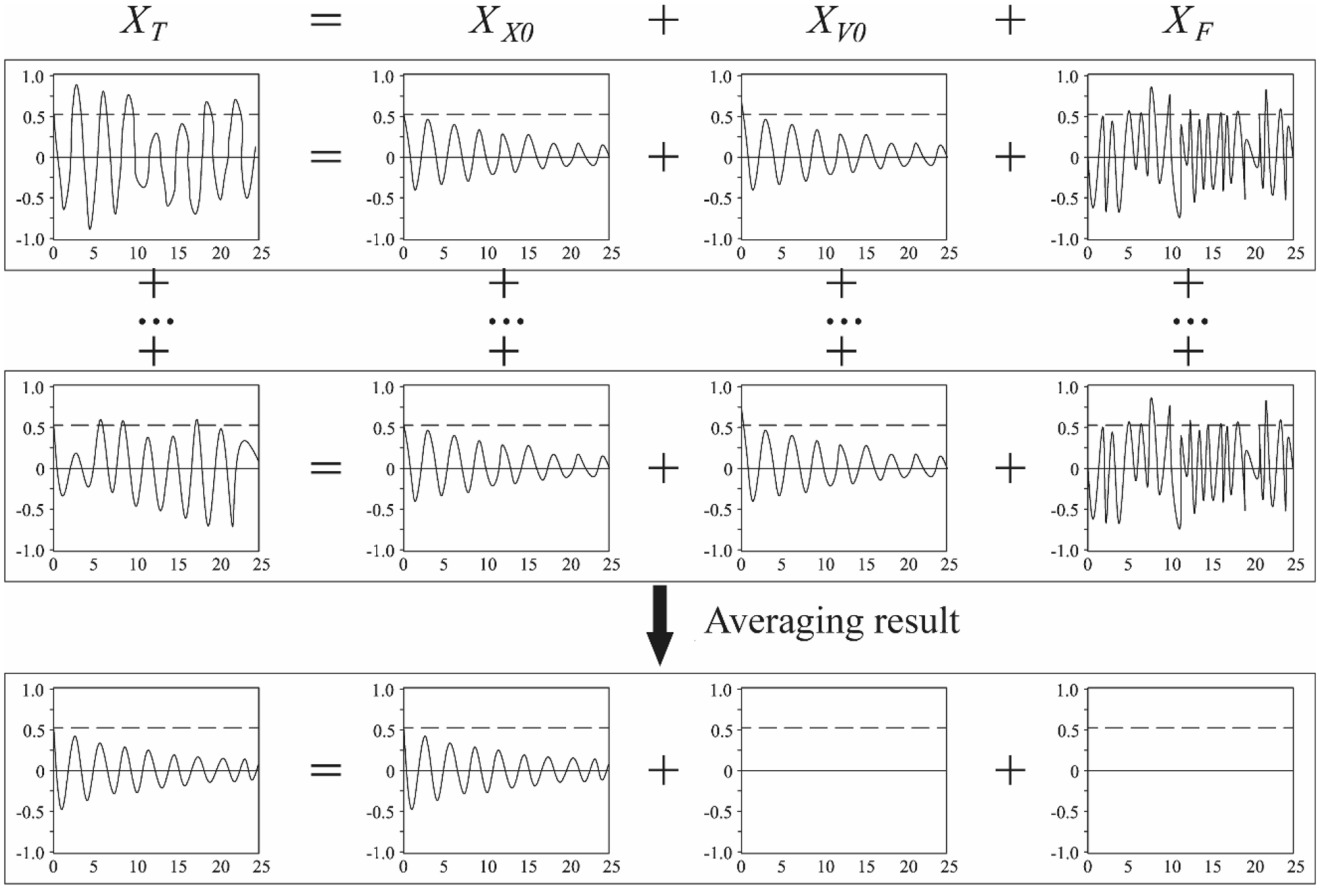
Conceptualization of RDT technique.

**Figure 6:**
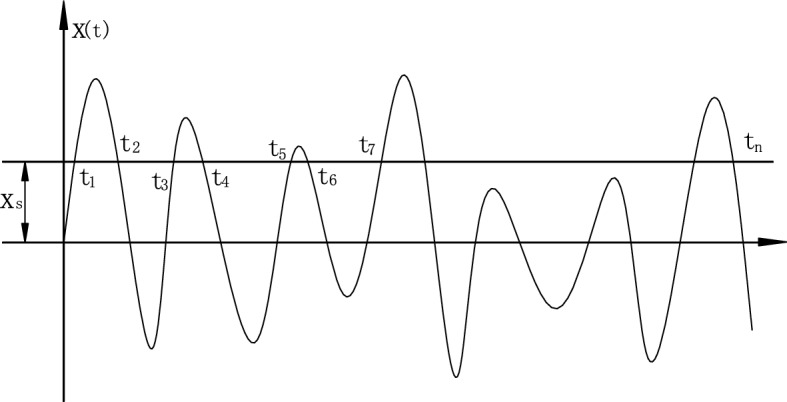
Cut out method for obtaining RDT curves.

Figure 5 shows the conceptualization of RDT and Fig. 6 shows the cut out method for obtaining RDT curves. If select appropriate threshold *Xs* to horizontally intercept the displacement time history *X*_*T*_ with sufficient length, and take each intersection point of the horizontal line X_S_ and the total displacement time history *X*_*T*_, that is, the displacement at each *t*_*i*_ moment, as the starting point. Then a series of displacement time histories with the same initial displacement, which are called RDT curves, can be obtained by intercepting a certain length of displacement time series to the right. If more than 500 groups of displacement time histories with the same initial displacement are superposed and averaged, the response components caused by initial velocity and external force are averaged to zero. Then a random decrement curve that begins to decay from the set threshold Xs can be obtained, which is a free decay curve that is close to the logarithmic law. The damping of this random decrement curve is the total damping of the structure, which can be obtained by Hilbert transform.

### Analysis of vibration modes

To identify the vibration modes of the ice-coated transmission line model, the theoretical analysis method is used to obtain the modal vibration frequencies of the transmission line.

For a horizontal suspension structure with both ends fixed at the same height, the vibration can be decomposed into the out-of-plane and in-plane directions. The in-plane vibration can be divided into antisymmetric and symmetric modes^32,33^. It is generally believed that vibration of in-plane antisymmetric mode will not produce additional tension in the transmission line. While for the in-plane symmetric mode of higher modal orders (excluding the first-order mode), it will produce an additional tension, which should be considered into the modal results. When the in-plane and out-of-plane modal frequencies are close, coupling between the in-plane and out-of-plane modes will occur, which would lead to multiple internal resonances simultaneously^34,35^.

According to 29 and 33, the out-of-plane vibration, the vibration frequencies and modes of the ice-coated transmission line model can be determined by the Eqs. (4) and (5):

4 $$f_{y,n} = \frac{n}{2l}\sqrt{\frac{H}{m}} , \, n = 1,2,3,...$$

5 $$\varphi_{n} (x) = A_{n} \sin \left( {\frac{n\pi x}{l}} \right), \, n = 1,2,3,...$$

where *n* represents the modal order; *m* is the total mass of the ice-coated transmission line model per unit length; *l* is the span; *H* is the horizontal component of the tension of the static transmission line model, which can be determined by Eq. (6); *h* is the sag of the transmission line; *g* is the gravitational constant. For this study, *m* = 0.34 kg/m, *l* = 3.4 m, and *h* = 0.4 m. As a result, *H* = 11.92N is calculated.

6 $$H = \frac{{mgl^{2} }}{8h}$$

For the in-plane antisymmetric vibration mode of the transmission line, which will not produce additional dynamic tension, the resulting vibration frequencies and modal functions can be expressed by the following formulas:

7 $$f_{z,2n} = \frac{n}{l}\sqrt{\frac{H}{m}} , \, n = 1,2,3,...$$

8 $$\varphi_{2n} (x) = A_{n} \sin \left( {\frac{2n\pi x}{l}} \right), \, n = 1,2,3,...$$

For the in-plane symmetric vibration mode, an additional cable tension is considered, the vibration frequencies and modal functions are expressed by the following formulas:

9 $$\tan \left( {\frac{{f_{z,n} l}}{{4\pi (H/m)^{2} }}} \right) = \frac{{f_{z,n} l}}{{4\pi (H/m)^{2} }} - \frac{4}{{\lambda^{2} }}\left( {\frac{{f_{z,n} l}}{{4\pi (H/m)^{2} }}} \right)^{3} , \, n = 1,3,5,...$$

10 $$\lambda^{2} = \left( \frac{mgl}{H} \right)^{2} {l \mathord{\left/ {\vphantom {l {\left( {\frac{{HL_{e} }}{{EA_{0} }}} \right)}}} \right. \kern-0pt} {\left( {\frac{{HL_{e} }}{{EA_{0} }}} \right)}}{ = }\left( \frac{8h}{l} \right)^{2} {l \mathord{\left/ {\vphantom {l {\left( {\frac{{HL_{e} }}{{EA_{0} }}} \right)}}} \right. \kern-0pt} {\left( {\frac{{HL_{e} }}{{EA_{0} }}} \right)}}$$

11 $$L_{e} = l \cdot \left[ {1 + 8\left( \frac{h}{l} \right)^{2} } \right]$$

Take the investigated model with a sag of 0.4 m as an example, *L*_*e*_ = 3.53 m, *λ*^2^ > 256π^2^. The analytical and test results of frequencies for the mid-span measurement point D_2_ are shown in Table 3. The test results are identified from the wind-induced response data at a wind speed of 4 m/s of 0° wind direction case. It can be seen that the values of the frequencies obtained by analytical method and identified by wind tunnel test are similar. Nevertheless, the differences are owing to the uneven quality of the ice-coated model during the model manufacturing process. Moreover, under the action of wind load, the axial tension of the transmission line is different from the static state, which will also cause errors.

**Table 3:** Modal frequency based on the measurement of point D_2_.

Motion component	Vibration frequency	Frequency /Hz				Modal shape
		Analytical results	Test results			
			0°	15°	30°	
Out-of-plane	*f*_*y,*1_	0.88	0.81	0.83	0.82	One loop
	*f*_*y,*2_	1.75	1.56	1.63	1.60	Two loops
	*f*_*y,*3_	2.63	2.38	2.42	2.34	Three loops
	*f*_*y,*4_	3.50	3.17	3.38	3.32	Four loops
	*f*_*y,*5_	4.38	4.07	–	3.93	Five loops
In-plane	Anti-symmetry *f*_*z,*2_	1.75	1.56	1.63	1.60	Two loops
	Anti-symmetry *f*_*z,*4_	3.50	3.17	3.38	3.32	Four loops
	Symmetric *f*_*z,*1_	2.51	2.38	2.42	2.34	One loop
	Symmetric *f*_*z,*3_	4.31	4.07	–	3.93	Three loops

## Statistical and spectral characteristics of aero-elastic responses

### Statistical characteristics analysis

**Figure 7:**
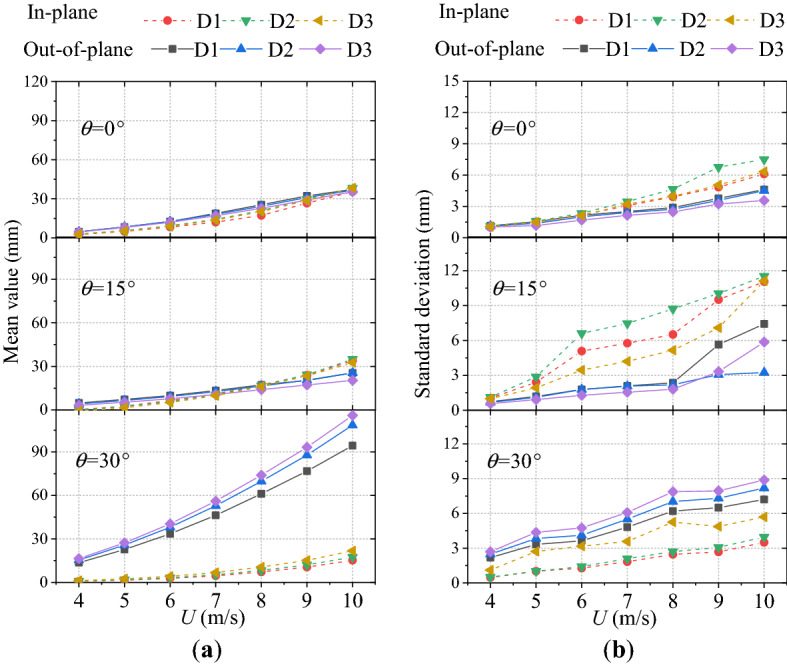
Mean and standard deviation values of wind-induced displacement with various wind speeds: (**a**) mean values of wind-induced displacement. (**b**) standard deviation values of wind-induced displacement.

The mean and standard deviation values of wind-induced displacements in various wind directions at different freestream velocities are shown in Fig. 7. It is observed that, for the direct flow (0°), the time-averaged deformation of each measuring point in the out-of-plane direction is close to or slightly larger than that in the in-plane direction. However, the variation characteristics of amplitude with the increment of the wind speed are obviously different from those of the time-averaged deformation. Specifically, when the wind speed exceeds a certain value (for example: 7 m/s in Fig. 7b), the in-plane amplitude of the ice-coated transmission line model tends to be significantly larger than the out-of-plane amplitude. And, it even reaches twice of the out-of-plane amplitude. For an oblique flow case of 15°, the increment of the in-plane average deformation and amplitude with increasing wind speed is greater than that in the out-of-plane direction. When it exceeds 5 m/s, the in-plane amplitude increases tremendously with wind speed. In the subsequent wind speed case, the maximum in-plane amplitude is three times of the out-of-plane amplitude, and twice that of the in-plane amplitude of direct flow case under the same conditions. When the wind direction is 30°, the time-averaged deformation and amplitude in the out-of-plane are much larger than those in the in-plane direction. In the whole wind speed range, the time-averaged deformation in the out-of-plane direction is ten times of the maximum in the in-plane direction at the same wind speed. Moreover, the maximum amplitude in out-of-plane is twice that of the in-plane amplitude. Here, the maximum amplitude is the maximum displacement minus the average displacement ($\overline{d}_{i}$); the root mean square is the standard deviation of the fluctuating displacement, which is denoted as $\sigma_{i}$. The evaluation methods are displayed as the Eqs. (1) and (2) in this paper.

### Spectral characteristics analysis

The normalized power spectral density (PSD) curves of wind-induced displacement responses at different wind directions are calculated and analyzed.

**Figure 8:**
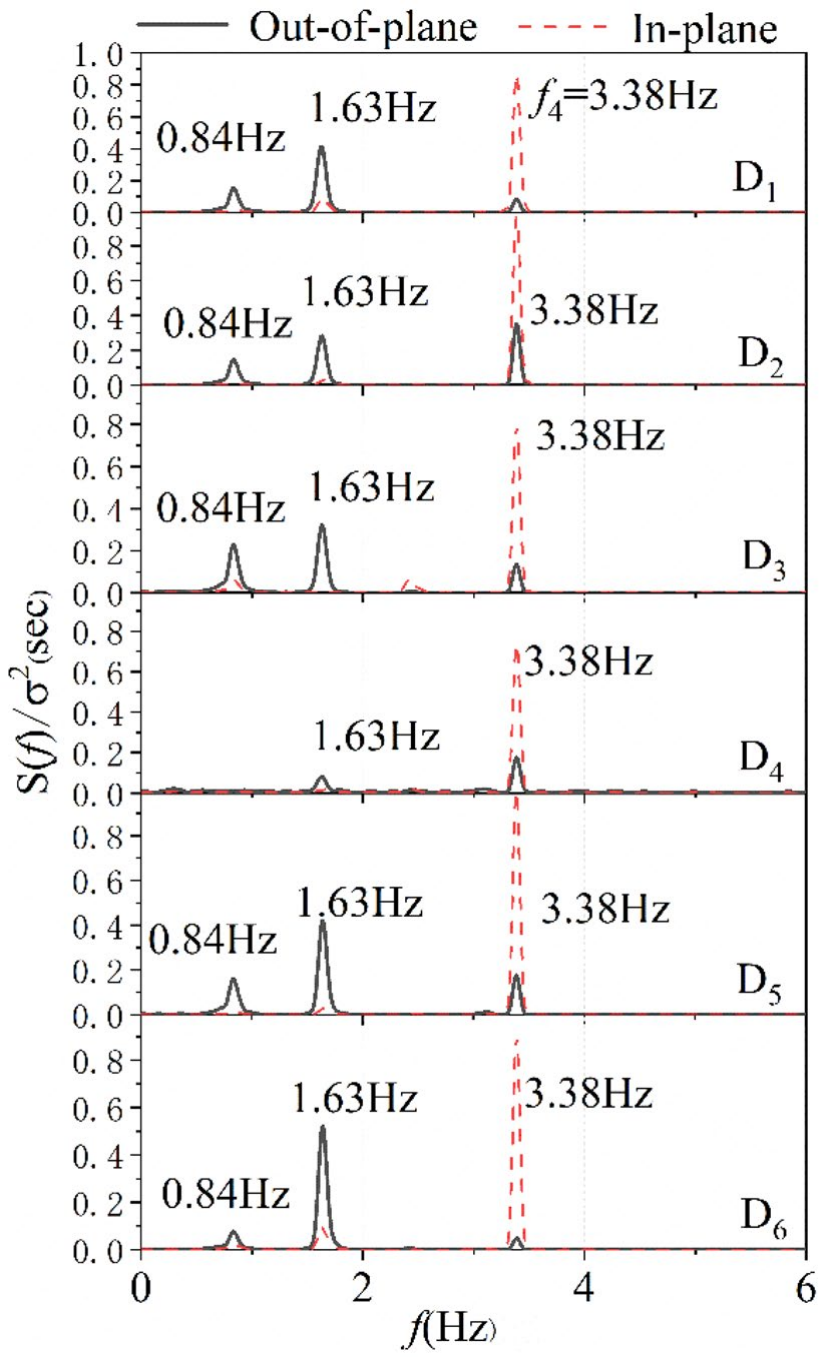
The PSD curves of wind-induced displacement responses at different measurement points when at wind speed of 6 m/s at wind direction of 15°.

**Figure 9:**
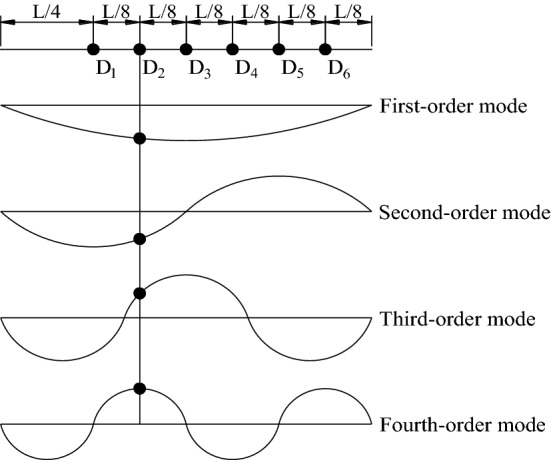
The first 4 order modal shapes and measuring points positions.

Figure 8 is the PSD curves of wind-induced displacement responses at different measurement points at wind speed of 6 m/s at the wind direction of 15°. It can be seen from the figure that the peak frequencies of power spectrum at different measuring points are basically the same. Considering that under various cases, the vibration of the transmission line is dominated by the first four modes, and the position of the measuring point D_2_ is not at the node position of the first four modes as shown in Fig. 9, and the number of modes captured in the test is the most comprehensive. Therefore, the test result of measurement point D_2_ is selected as the main analysis object of study.

**Figure 10:**
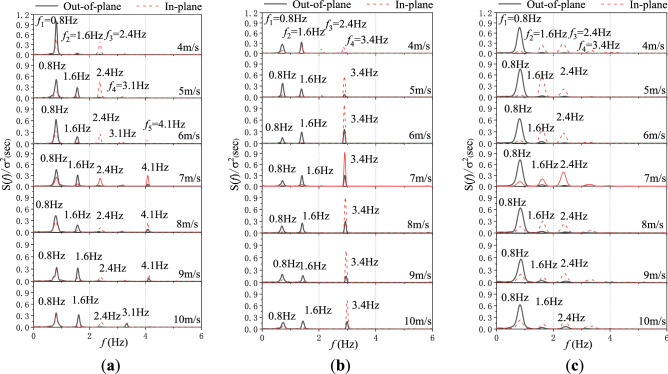
The PSD curves of wind-induced displacement responses: (**a**) 0°wind direction. (**b**) 15°wind direction. (**c**) 30°wind direction.

As illustration, the PSD curves of the measurement mid-span point D_2_ are shown in Fig. 10, where *S*(*f*)/*σ*^2^ represents the normalized PSD of the wind-induced displacement, *f*_*j*_ (*j* = 1, 2, 3, 4 …) represent the *j*th modal vibration frequencies identified from the experimental data.

It can be observed from Fig. 10a that for the direct flow of 0°, the fundamental modal frequencies of the out-of-plane displacements are exactly the same at 0.8 Hz. For the oblique flow case of 15°, obvious spectral peaks can be observed in Fig. 10b around the 4th order mode vibration at 3.4 Hz, which appear in both the out-of-plane and the in-plane displacements simultaneously, when the wind speed exceeds 5 m/s. Compared with the 0° case, the component of the wind force in the axial direction of the ice-coated transmission line increases significantly at the 15° case, which affects the modal frequency of the conductor to excite a 3.4 Hz in-plane vibration mode. The modal frequency is the close to the modal frequency of the out-of-plane vibration, resulting in a coupling vibration between in-plane and out-of-plane directions. When the wind direction is 30°, as shown in Fig. 10c, in the out-of-plane direction, the 1st mode vibration mode becomes dominant. The modes of the 1st and 3rd orders are excited simultaneously. In the in-plane direction, the 3rd mode becomes the dominant. It can be seen that the dominant frequencies and modes are different at 15° and 30° wind directions.

According to the in-plane and out-of-plane resonance mechanisms^32^, the lowest two in-plane and out-of-plane modes are not involved in an internal resonance with any of the other modes. Therefore, For the direct flow of 0°, the coupling resonance will not occur in the 1st mode. For the oblique flow case of 15°, it will induce a 1:1 internal resonance for the 4th order mode vibration at 3.4 Hz in both the out-of-plane and in-plane directions. When the wind direction is 30°, there will be not any 1:1, 1:2, or 1:3 internal resonance between the 1st order mode vibration in the out-of-plane direction and the first three orders modes vibration in the in-plane direction. However, there might be a 1:1 internal resonance between the 3rd order mode vibration in the out-of-plane and in-plane directions.

## Galloping characteristics analysis

In this section, we will further analyze the galloping characteristics and determine whether the coupling vibration occurs based on the vibration trajectories and damping ratios at different situations.

### Vibration trajectory

In this section, the galloping characteristics of transmission lines will be further analyzed based on the vibration trajectories.

**Figure 11:**
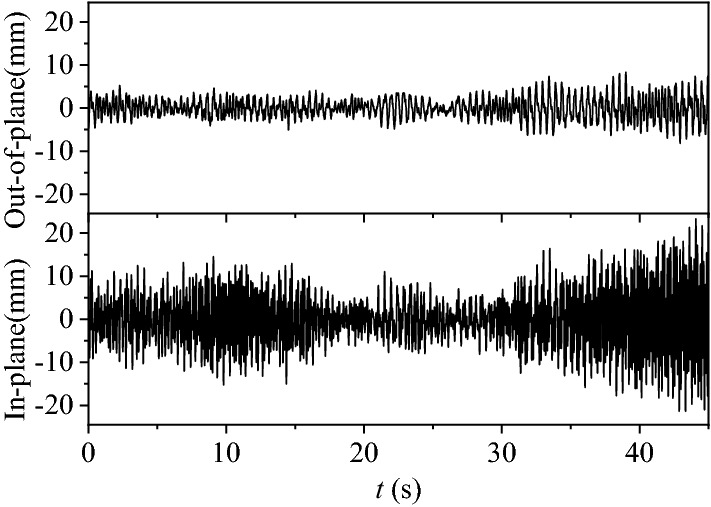
The time histories of fluctuating wind-induced displacement for measurement point D_2_ at a velocity of 6 m/s (15° wind direction).

Figure 11 shows the time histories of fluctuating displacement for measurement point D_2_ at 15° wind direction when the wind speed is 6 m/s (the mean value of the displacement has been subtracted). The results show that the displacement time histories diverge gradually, which indicates a galloping phenomenon. Combined with the analysis for Fig. 10b, it is concluded that, the galloping in this condition may be caused by the in-plane and out-of-plane resonance of the 4th order modes with the frequency ratio of 1:1.

**Figure 12:**
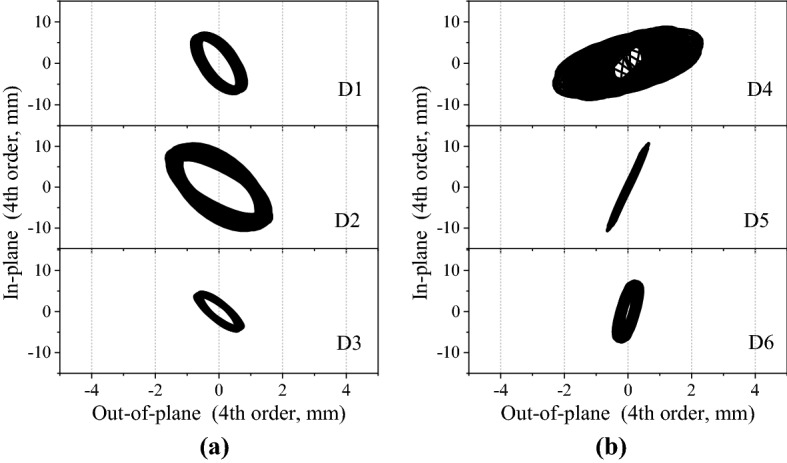
The vibration trajectories for all the measurement points at 15° wind direction when wind speed is 6 m/s: (**a**) measurement points D_1_, D_2_, and D_3_, (**b**) measurement points D_4_, D_5_, and D_6_.

Figure 12 shows the vibration trajectories of the in-plane and out-of-plane trajectories of the 4th order modal for measurement points D_1_ ~ D_6_ at 15° wind direction when the wind speed is 6 m/s. The filter band was 3.2 ~ 4.0 Hz. The results show that the all the trajectories of the 4th order modal displacements of the six measurement points are elliptical.

**Figure 13:**
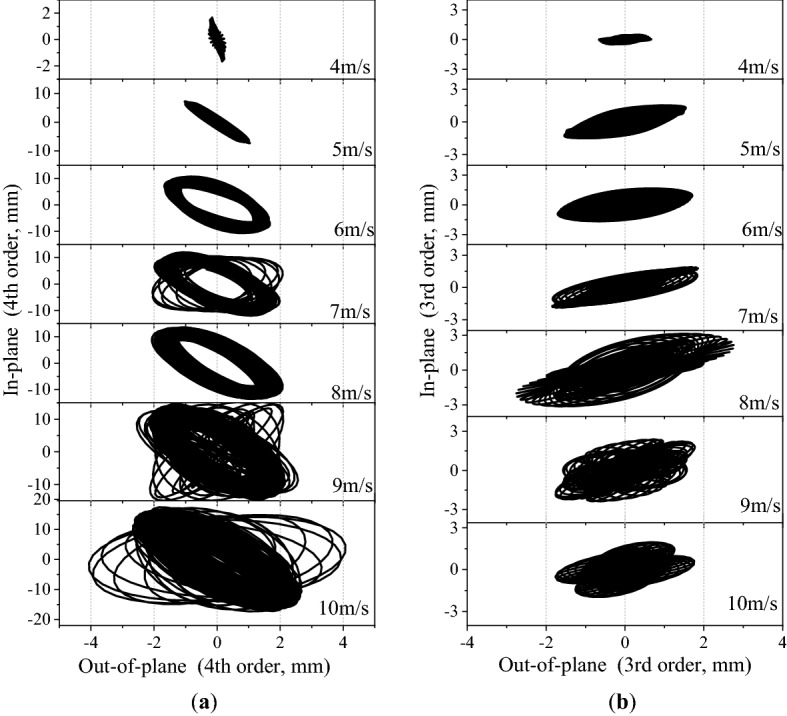
The vibration trajectories for measurement point D_2_ at different wind speeds and directions: (**a**) 15° wind direction. (**b**) 30° wind direction.

Figure 13 shows the vibration trajectories of the in-plane and out-of-plane displacements for measurement point D_2_ at different wind speeds and directions. Figure 13a shows the vibration trajectory at 15° wind direction at different wind speeds. When the wind speed exceeds 5 m/s, the vibration trajectory in 3.4 Hz frequency mode shows an obvious elliptical shape. The diameter of the elliptical trajectory increases with the wind speed. Figure 13b shows the vibration trajectory of the 3rd order mode vibration at different wind speeds of 30°. It can be seen that the vibration trajectories are all elliptical at different wind speed. However, the lengths of the elliptical trajectories are much smaller than that of the 15° wind direction cases as shown in Fig. 13a.

According to the spectral analysis in the previous section, it can be determined that the coupling resonance between the in-plane and out-of-plane vibrations does not occur in the 0° wind direction. At the wind direction of 15°, when the wind speed exceeds 5 m/s, the ice-coated transmission line model gallops. At the wind direction of 30°, when the wind speed exceeds 8 m/s, there may be a 1:1 internal resonant between the 3rd order mode vibrations in the out-of-plane and in-plane directions.

### Damping ratio

According to the in-plane and out-of-plane resonance mechanisms^32^, the lowest two in-plane and out-of-plane modes are not involved in an internal resonance with any of the other modes. Therefore, the damping ratio of the in-plane displacement and out-of-plane displacement time history of the third-order and fourth-order modes is a basis for determining whether internal resonance occurs. Using Random Decrement Technique (RDT)^31^, the 4th modal damping ratios are obtained based on the displacement time history of the mode corresponding to the frequency of 3.4 Hz at the 15° wind direction. The filter band was taken as 3.2 ~ 4.0 Hz. The resulting damping ratios of out-of-plane and in-plane vibrations *ζ*_4*y*_ and *ζ*_4*z*_ are shown in Table 4. It is observed that when the wind speed is greater than or equal to 5 m/s, the damping ratios are all negative.

**Table 4:** Damping ratios of the 4th mode for 15° wind direction.

Wind speed (m/s)	*ζ*_4*y*_	*ζ*_4*z*_
4	0.160	0.037
5	− 0.039	− 0.047
6	− 0.013	− 0.014
7	− 0.006	− 0.005
8	− 0.014	− 0.011
9	− 0.010	− 0.022
10	− 0.004	− 0.006

## Conclusions

This paper investigates the galloping characteristics of ice-coated transmission lines in oblique flows. Firstly, the aero-elastic wind-induced responses of the fan-shaped ice-coated transmission lines are measured in a series of wind tunnel tests. Subsequently, the influences of oblique flows on the statistical and spectral characteristics of wind-induced responses are analyzed. Finally, the galloping characteristics including vibration trajectory and damping ratio are analyzed. The following concluding remarks are drawn:

1. Galloping of the ice-coated transmission lines is more likely to occur in the oblique flows (15° and 30°) than in the direct flow (0°).
2. For the direct flow case (0°), no notable galloping phenomenon was observed. The in-plane vibration was dominant, which reached about twice of the out-of-plane vibration amplitude. Multiple vibration modes were observed in both the out-of-plane and in-plane directions.
3. For the oblique flow case of 15°, at wind speeds over 5 m/s, a galloping characterized by elliptical trajectories was observed. The maximum in-plane amplitude is three times of the out-of-plane amplitude, and twice of the in-plane amplitude of the direct flow (0°) under the same conditions.
4. For the oblique flow case of 30°, elliptical trajectories were observed in the entire wind speed range. The maximum amplitude in out-of-plane is twice of the in-plane amplitude, and the in-plane amplitude reached twice of that in the direct wind flow (0°) at the same wind speed.
5. Based on the above findings, it is suggested that, in the plan of transmission line routes, the intersection angle between the line direction and the winter monsoon direction should be avoided between 15° and 30°. If it is inevitable, appropriate anti-galloping devices is highly recommended to be installed on the lines.

The results in this study can also be used to validate the numerical simulations on the fluid–structure interaction analysis of ice-coated transmission lines.

## Data Availability

The datasets used and analyzed during the current study available from the corresponding author on reasonable request.
